# Supplementary material for: Aerobic Exercise Attenuates Doxorubicin-Induced Cardiomyopathy by Suppressing NLRP3 Inflammasome Activation in a Rat Model
Source: Int J Mol Sci. 2024 Sep 7;25(17):9692. doi: 10.3390/ijms25179692 (PMC11395441; doi:10.3390/ijms25179692)
Supplement: Supplementary file 1 [file ijms-25-09692-s001.zip › ijms-3187921-supplementary.pdf]

SUPPLEMENTARY FIGURE

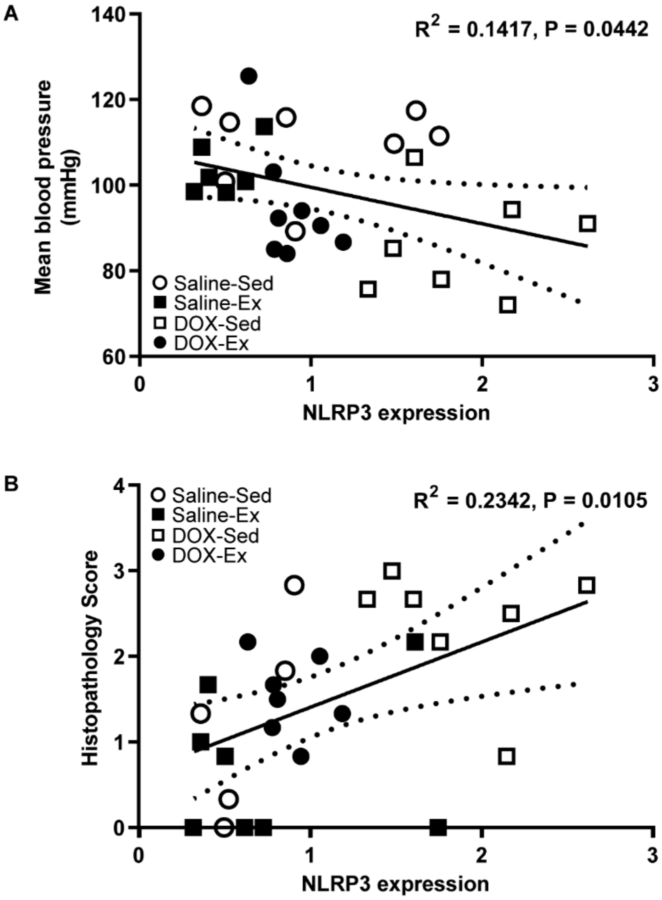

Figure S1. Relationship between mean arterial blood pressure and histopathology score to the cardiac expression levels of NLRP3 from all experimental group combinations. Linear regression analysis indicated significant relations were found between NLRP3 expression and mean blood pressure (A; $R^2 = 0.1417$) or histopathological score (B; $R^2 = 0.2342$). R^2 represented the coefficient of determination. R represented Pearson correlation coefficient, with R values of 0–0.3, 0.3–0.5 and > 0.5 indicating weak, moderate, and strong correlation, respectively.
